# Supplementary material for: Zoonotic Streptococcus imports glucose to inhibit stringent response and promote growth during meningitis
Source: Nat Microbiol. 2025 Dec 15;11(1):125–41. doi: 10.1038/s41564-025-02194-2 (PMC12768970; doi:10.1038/s41564-025-02194-2)
Supplement: Supplementary file 1 — Reporting Summary [file 41564_2025_2194_MOESM1_ESM.pdf]

nature portfolio

Corresponding author(s): Zhe Ma
Last updated by author(s): Oct 5, 2025

Reporting Summary

Nature Portfolio wishes to improve the reproducibility of the work that we publish. This form provides structure for consistency and transparency in reporting. For further information on Nature Portfolio policies, see our Editorial Policies and the Editorial Policy Checklist.

Statistics

For all statistical analyses, confirm that the following items are present in the figure legend, table legend, main text, or Methods section.

n/a	Confirmed	
☐	☒	The exact sample size (*n*) for each experimental group/condition, given as a discrete number and unit of measurement
☐	☒	A statement on whether measurements were taken from distinct samples or whether the same sample was measured repeatedly
☐	☒	The statistical test(s) used AND whether they are one- or two-sided<br>*Only common tests should be described solely by name; describe more complex techniques in the Methods section.*
☒	☐	A description of all covariates tested
☐	☒	A description of any assumptions or corrections, such as tests of normality and adjustment for multiple comparisons
☐	☒	A full description of the statistical parameters including central tendency (e.g. means) or other basic estimates (e.g. regression coefficient) AND variation (e.g. standard deviation) or associated estimates of uncertainty (e.g. confidence intervals)
☐	☒	For null hypothesis testing, the test statistic (e.g. *F*, *t*, *r*) with confidence intervals, effect sizes, degrees of freedom and *P* value noted<br>*Give P values as exact values whenever suitable.*
☒	☐	For Bayesian analysis, information on the choice of priors and Markov chain Monte Carlo settings
☒	☐	For hierarchical and complex designs, identification of the appropriate level for tests and full reporting of outcomes
☒	☐	Estimates of effect sizes (e.g. Cohen's *d*, Pearson's *r*), indicating how they were calculated

Our web collection on statistics for biologists contains articles on many of the points above.

Software and code

Policy information about availability of computer code

Data collection	The following software was used to collect data:<br>Image Lab (v 6.0.1);<br>ZEISS software Zen (v 3.9.101.0000);<br>Berthold Technologies software IndiGo in vivo imaging system (v2.0.2.0);<br>illumina NextSeq 500/550 Control Software;<br>Nano Drop 2000/2000c (v 1.6.198);<br>Tecan i-control (v 3.9.1.0);<br>StepOne Software (v 2.3);<br>Amersham Typhoon software CytExpert (v 2.3);
Data analysis	The following software and code was used to collect data:<br>GraphPad Software 9.0;<br>R Studio (v1.4.1717);<br>ImageJ Software (v2.14.0);<br>SnapGene Software(v4.3.4);<br>Circular-Plot Software(v18.2.0);<br>Biotie2 (v2.2.5);<br>FeatureCounts (v1.5.0);<br>RSEM (v1.2.12);<br>Phyper (v3.6.0);<br>MUSCLE (v3.8.31);

MMSeqs2 (15-6f452);
Blastn (v2.7.1, NCBI);
Jalview 2.11.4.1.

For manuscripts utilizing custom algorithms or software that are central to the research but not yet described in published literature, software must be made available to editors and reviewers. We strongly encourage code deposition in a community repository (e.g. GitHub). See the Nature Portfolio guidelines for submitting code & software for further information.

Data

Policy information about availability of data

All manuscripts must include a data availability statement. This statement should provide the following information, where applicable:

- Accession codes, unique identifiers, or web links for publicly available datasets
- A description of any restrictions on data availability
- For clinical datasets or third party data, please ensure that the statement adheres to our policy

The raw files for RNAseq data generated in this study have been deposited in the NCBI database under accession code BioProject ID PRJNA1131986 (https://www.ncbi.nlm.nih.gov/bioproject/PRJNA1131986).
The raw files for Tn-seq data generated in this study have been deposited in the NCBI database under accession code BioProject ID PRJNA1132128 (https://www.ncbi.nlm.nih.gov/bioproject/PRJNA1132128).
The raw files for STAMP data generated in this study have been deposited in the NCBI database under accession code BioProject ID PRJNA1132155 (https://www.ncbi.nlm.nih.gov/bioproject/PRJNA1132155).
The scanned HE-stained slide files are available on Mendeley Data and can be downloaded from the following link: https://data.mendeley.com/datasets/fkg44432ct/1.
Source data are provided with this paper. The data that support the findings of this study are available from the corresponding author upon request.

Research involving human participants, their data, or biological material

Policy information about studies with human participants or human data. See also policy information about sex, gender (identity/presentation), and sexual orientation and race, ethnicity and racism.

Reporting on sex and gender	N/A
Reporting on race, ethnicity, or other socially relevant groupings	N/A
Population characteristics	N/A
Recruitment	N/A
Ethics oversight	N/A

Note that full information on the approval of the study protocol must also be provided in the manuscript.

Field-specific reporting

Please select the one below that is the best fit for your research. If you are not sure, read the appropriate sections before making your selection.

☒ Life sciences ☐ Behavioural & social sciences ☐ Ecological, evolutionary & environmental sciences

For a reference copy of the document with all sections, see nature.com/documents/nr-reporting-summary-flat.pdf

Life sciences study design

All studies must disclose on these points even when the disclosure is negative.

Sample size	There are at least three biological replicates in all of the experiments unless otherwise stated.No statistical methods were used to pre-determine sample sizes, but our sample sizes are similar to those reported in previous publications (Pletzer D, et al. mBio. 2017;8: e00140-17; Chang J, et al. Nat Commun. 2025;16: 6928z)
Data exclusions	No data were excluded.
Replication	All experiments were performed with at least three independent biological replicates and repeat at least for two times.
Randomization	The strains are separated to specific group according to their known single variables. Animals were randomly separated to different experimental groups. Microscopy observations were made from random selected vision, and multiple independent slices from different biological replications were taken for analysis.
Blinding	Data collection and analysis were not performed blind to the conditions of the experiments, except for histopathology scoring.

Reporting for specific materials, systems and methods

We require information from authors about some types of materials, experimental systems and methods used in many studies. Here, indicate whether each material, system or method listed is relevant to your study. If you are not sure if a list item applies to your research, read the appropriate section before selecting a response.

Materials & experimental systems

n/a	Involved in the study
☐	☒ Antibodies
☒	☐ Eukaryotic cell lines
☒	☐ Palaeontology and archaeology
☐	☒ Animals and other organisms
☒	☐ Clinical data
☒	☐ Dual use research of concern
☒	☐ Plants

Methods

n/a	Involved in the study
☒	☐ ChIP-seq
☒	☐ Flow cytometry
☒	☐ MRI-based neuroimaging

Antibodies

Antibodies used

Anti-SzM antibody was conducted by our lab in previous study (1:500 dilution).
Goat anti-mouse IgG H&L (Alexa Fluor® 488) (Abcam, ab150113, 1:1000 dilution).
Rabbit anti-prfA(crp) polyclonal antibody (CUSABIO,CSB-PA325257XA01LPY, Lot: K1231A, 1:2000 dilution)
Anti-GroEL antibody was conducted by our lab in previous study (1:1000 dilution).
Rabbit anti-ptsH(HPr) polyclonal antibody (CUSABIO,CSB-PA362538HA01BRJ, Lot: CBF0621A, 1:2000 dilution)
Goat Anti-Rabbit IgG H&L (HRP) (Abcam, ab97051, 1:5000 dilution)

Validation

Specificity of SzM antibody is validated in IFA test in figure 1c to verify the distribution of SEZ (DOI: 10.1128/spectrum.01742-22).
Rabbit anti-prfA (crp) polyclonal antibody: Used for the detection of intracellular Crp levels in Figure 6e.
GroEL antibody: Used as an internal control for bacterial protein expression in Figure 6e.
Rabbit anti-ptsH (HPr) polyclonal antibody: Used for the analysis of intracellular HPr phosphorylation levels in Figure 6e.

Animals and other research organisms

Policy information about studies involving animals; ARRIVE guidelines recommended for reporting animal research, and Sex and Gender in Research

Laboratory animals

All mice used in this study were female C57BL/6J mice (GemPharmatech, Nanjing) aged between 6 and 8 weeks. All swine used in this study to collected CSF were Landrace pigs (Zhao Fenghua biotechnology, Nanjing) aged between 12 and 16 weeks.

Wild animals

No wild animals were used.

Reporting on sex

In this study, only female mice were used to minimize variability and ensure consistency across all experimental conditions. Female mice were chosen for easy to operate. Using single sex reduced biological variability and helped to achieve more consistent and reliable results.

Field-collected samples

No field-collected samples were used.

Ethics oversight

All mice before experiment are housed in specific pathogen free conditions at Nanjing Agricultural University Laboratory Animal Center for 7 days prior to the experiment. All mice were kept under a 12-hour light-dark cycles, with temperature controlled at 22-24°C and humidity at 40-60%. Mice had free access to food and water. All animal experiments were performed with protocols approved by the Laboratory Animal Welfare and Ethics Committee of Nanjing Agricultural University (protocol number: NJAU.No20220311038) in accordance with the Laboratory Animal Guideline for ethical review of animal welfare (GB/T 35892-2018).

Note that full information on the approval of the study protocol must also be provided in the manuscript.

Plants

Seed stocks	N/A
Novel plant genotypes	N/A
Authentication	N/A
