# Supplementary material for: Zoonotic Streptococcus imports glucose to inhibit stringent response and promote growth during meningitis
Source: Nat Microbiol. 2025 Dec 15;11(1):125–41. doi: 10.1038/s41564-025-02194-2 (PMC12768970; doi:10.1038/s41564-025-02194-2)

Figure 6c

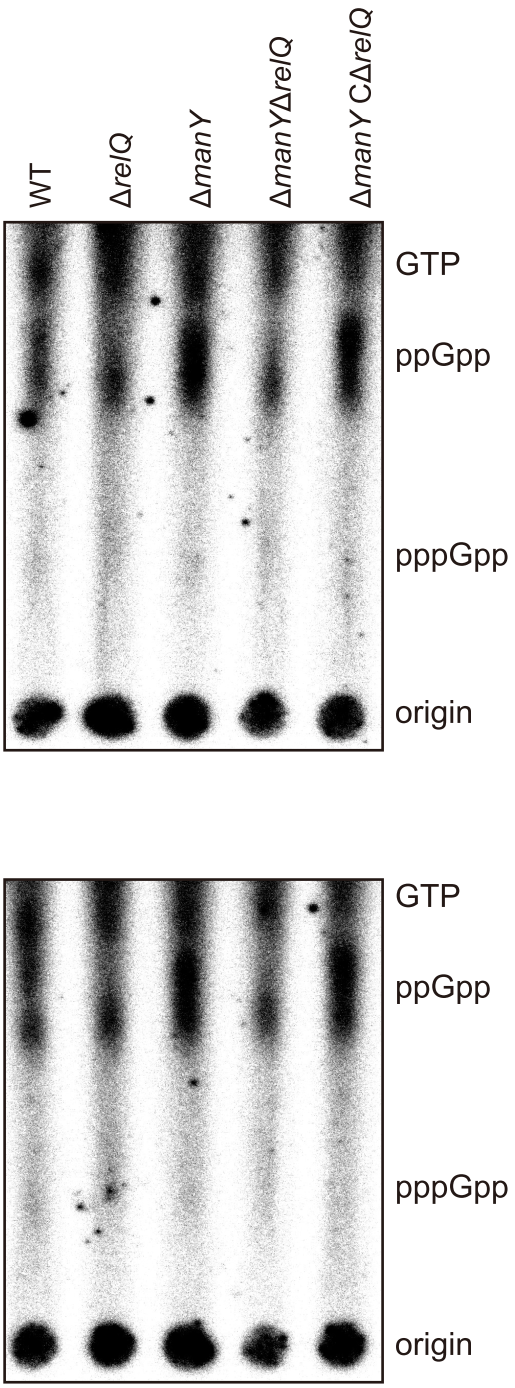
WT
ΔrelQ
ΔmanY
ΔmanYΔrelQ
ΔmanY CΔrelQ
GTP
ppGpp
pppGpp
origin
GTP
ppGpp
pppGpp
origin

Figure 6d

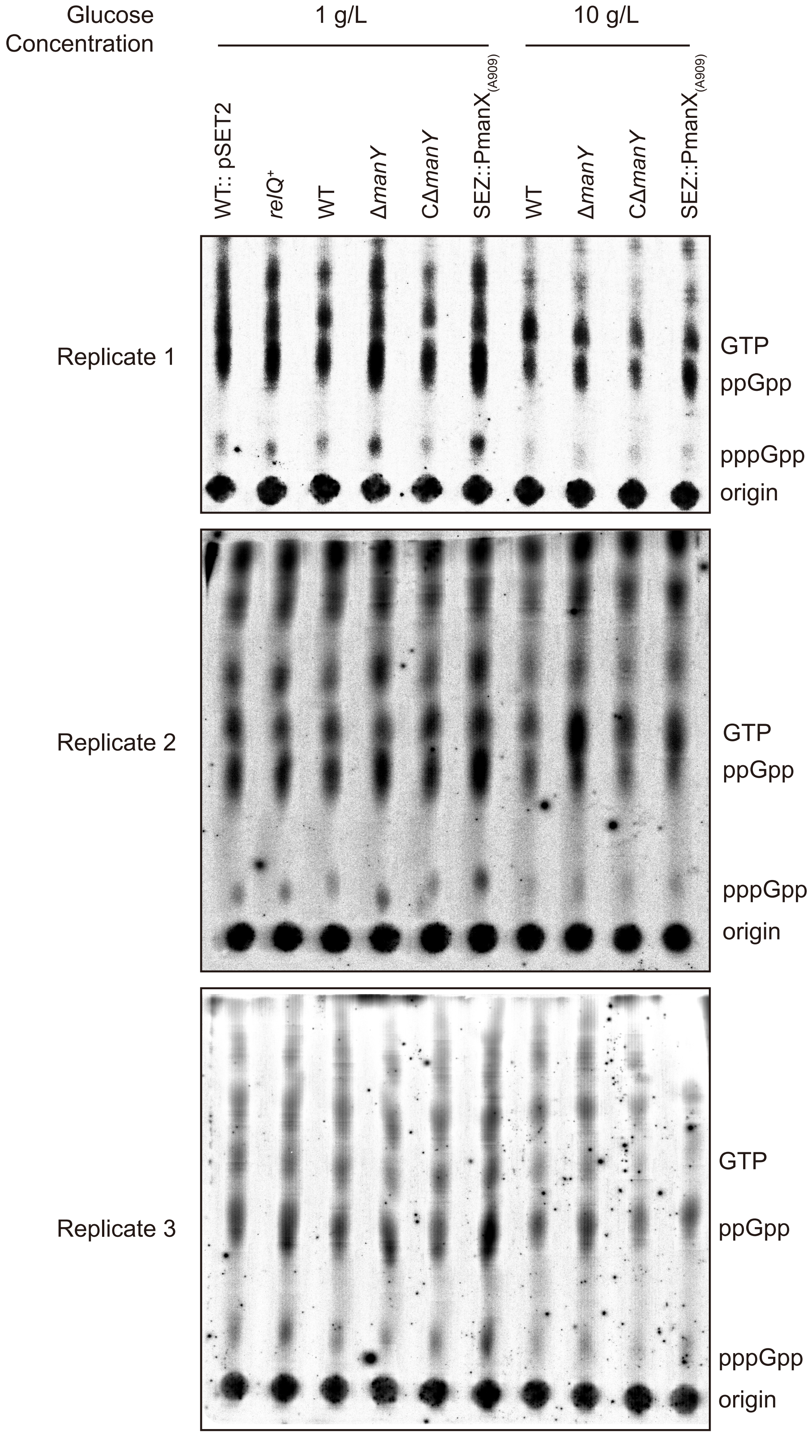
Glucose Concentration
1 g/L
10 g/L
WT:: pSET2
relQ+
WT
ΔmanY
CΔmanY
SEZ::PmanX(A909)
WT
ΔmanY
CΔmanY
SEZ::PmanX(A909)
Replicate 1
Replicate 2
Replicate 3
GTP
ppGpp
pppGpp
origin

Figure 6e Crp&GroEL Replicate#1

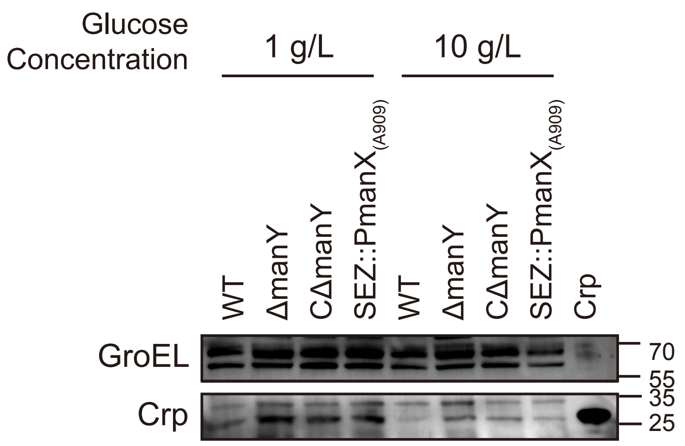

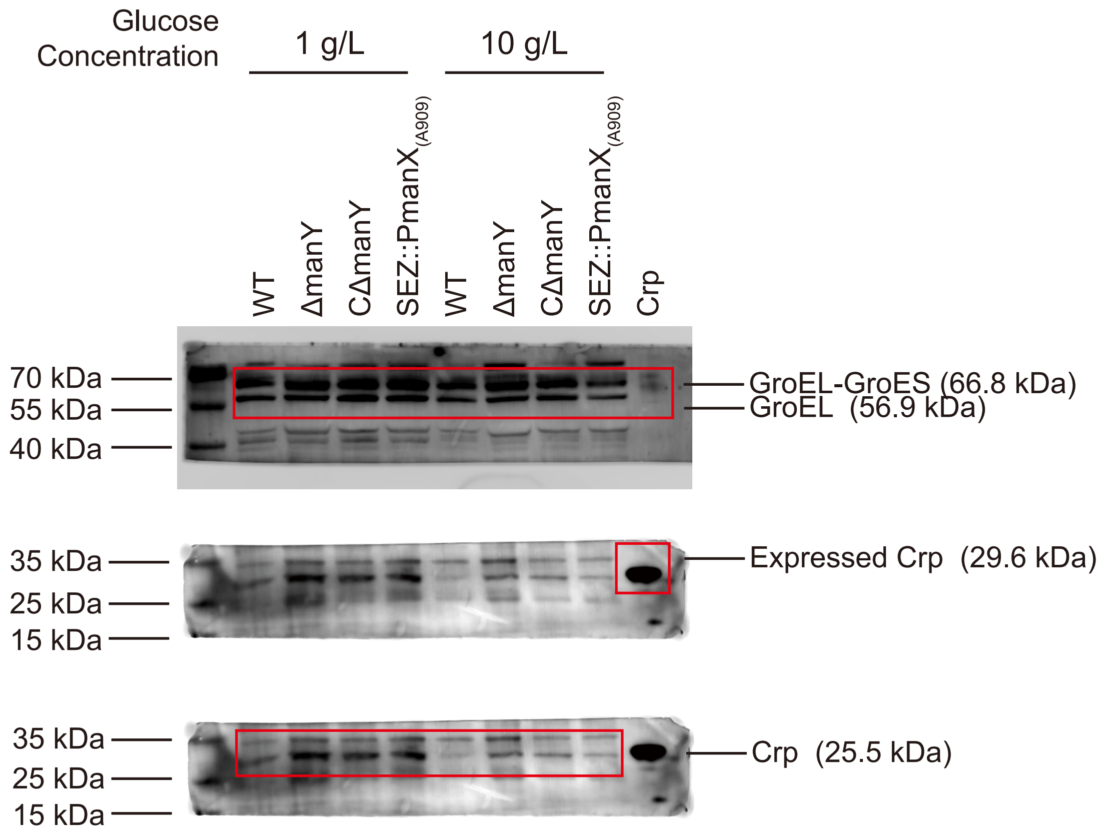

Figure 6e Crp&GroEL Replicate#2

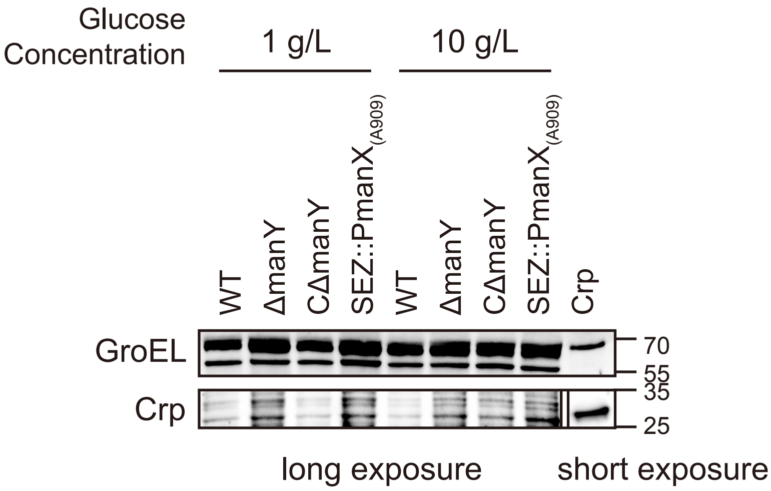

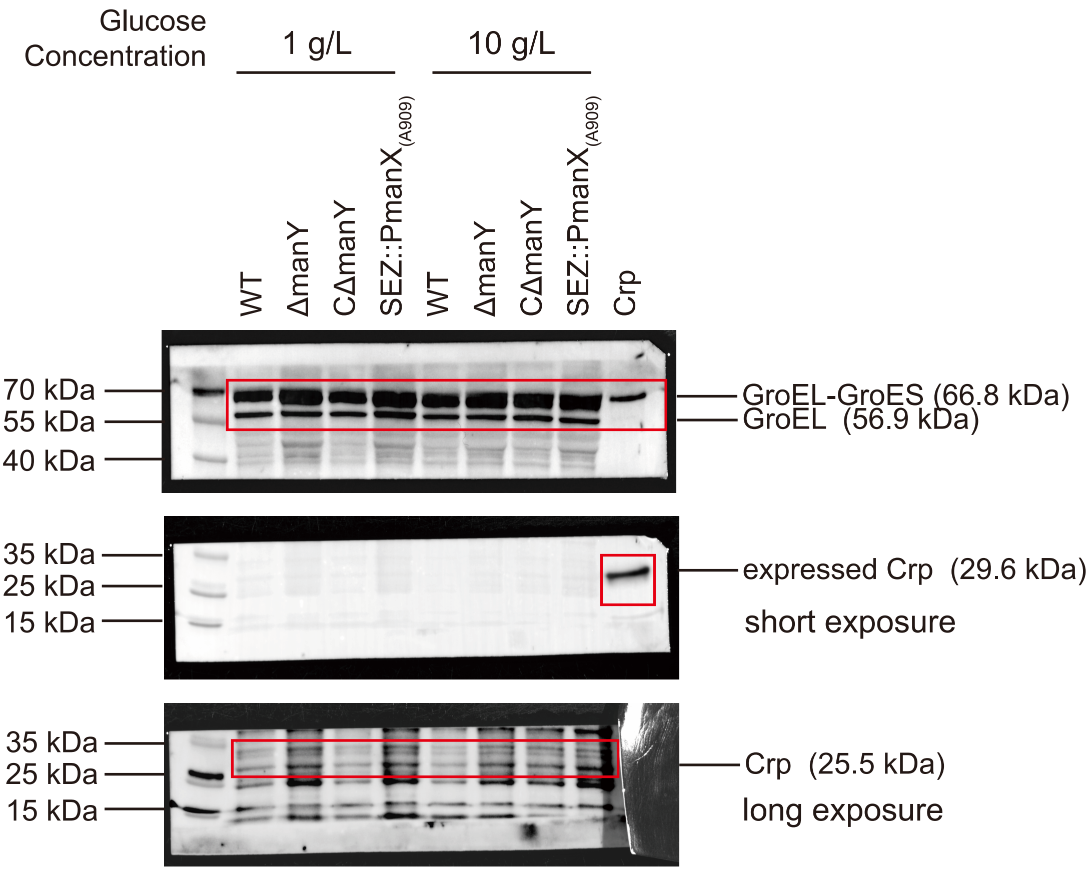

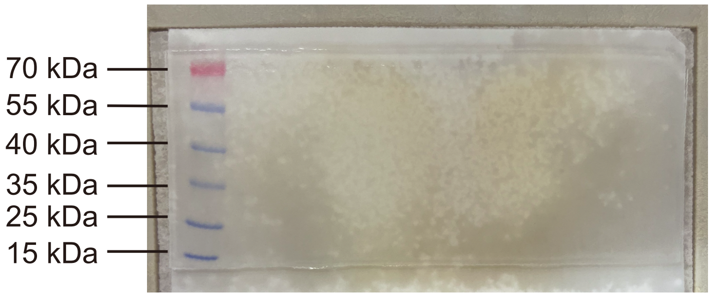

Figure 6e Crp&GroEL Replicate#3

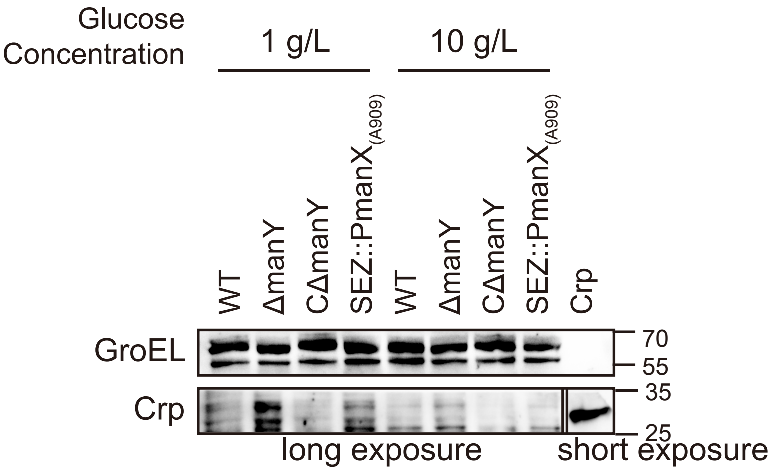

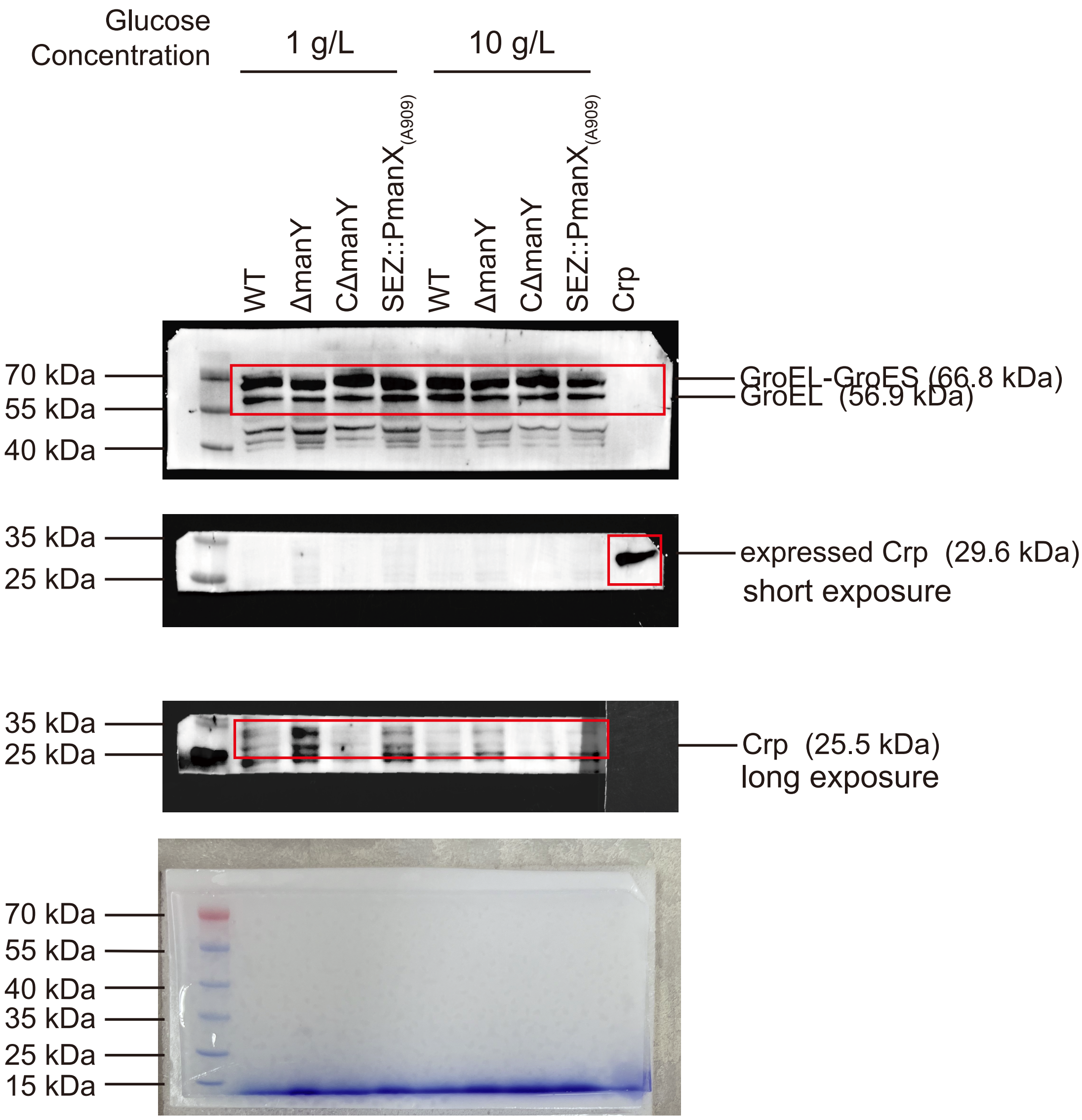

Figure 6e P-HPr

Replicate #1

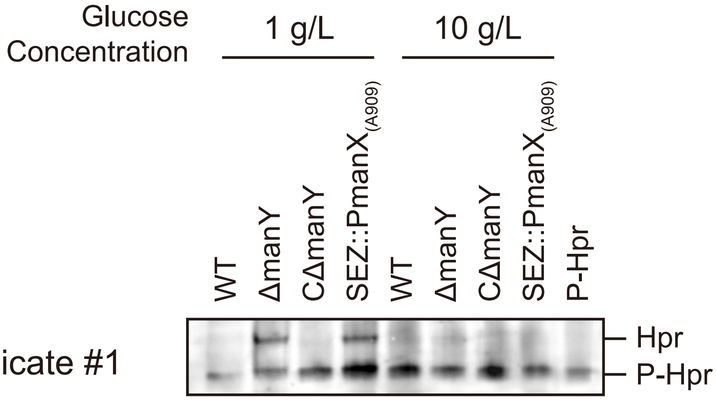

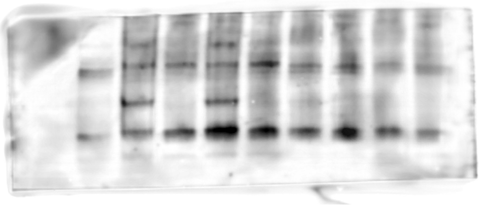

Replicate #2

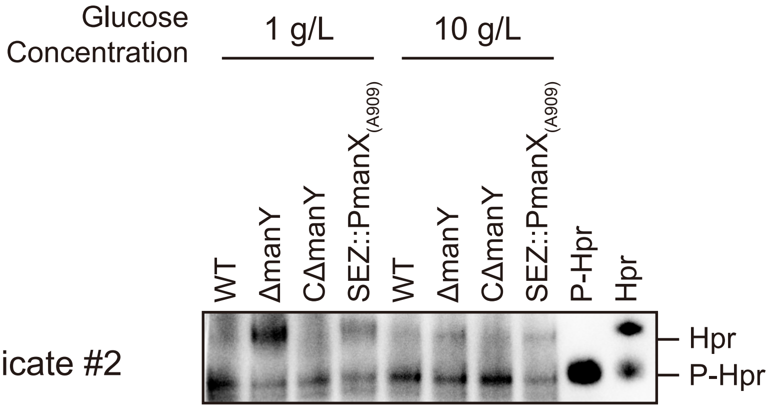

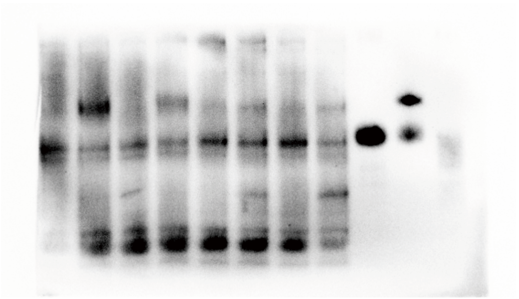

Replicate #3

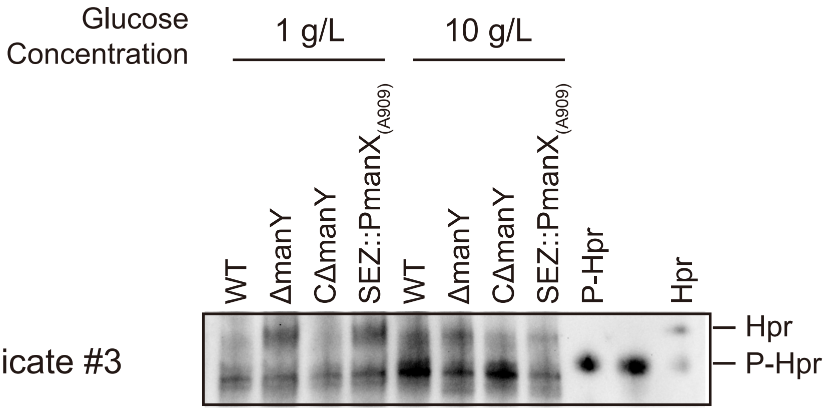

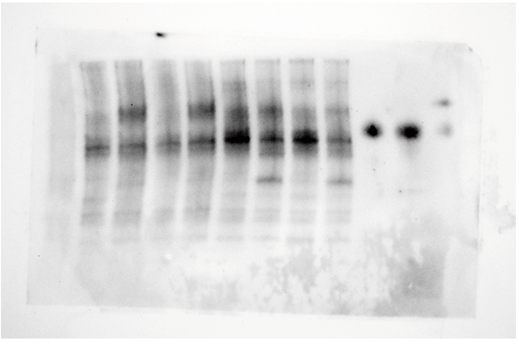

Figure 6f

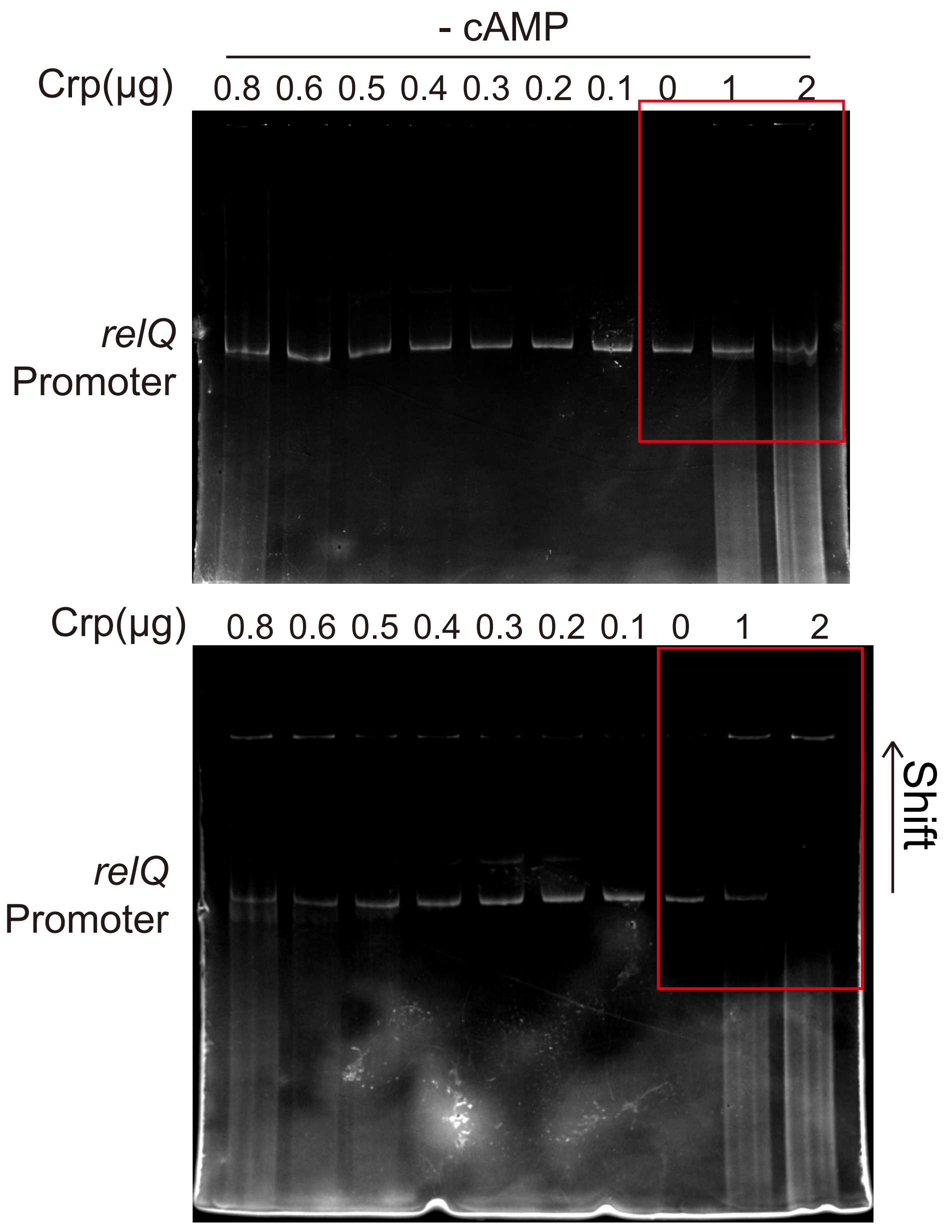

- cAMP
Crp(μg) 0.8 0.6 0.5 0.4 0.3 0.2 0.1 0 1 2
relQ
Promoter
Crp(μg) 0.8 0.6 0.5 0.4 0.3 0.2 0.1 0 1 2
relQ
Promoter
Shift

Figure 6i

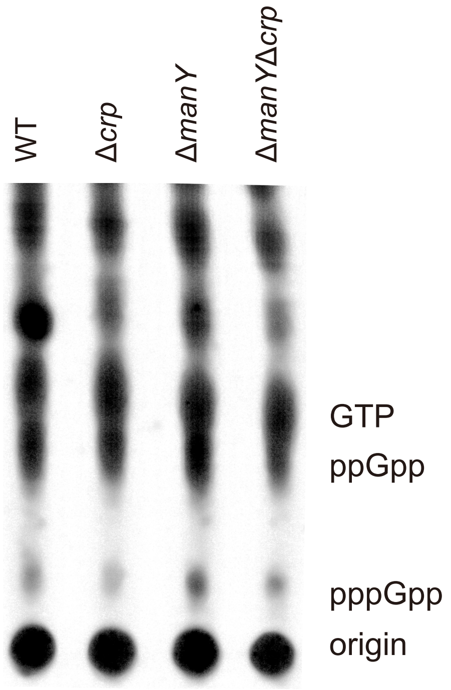
WT
Δcrp
ΔmanY
ΔmanYΔcrp
GTP
ppGpp
pppGpp
origin

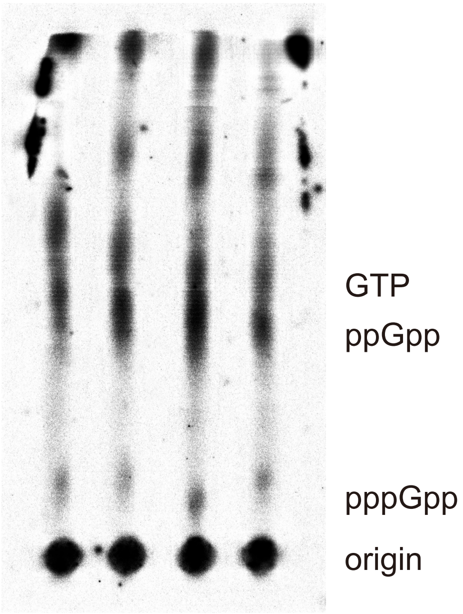
GTP
ppGpp
pppGpp
origin

Extended Data Figure 6b

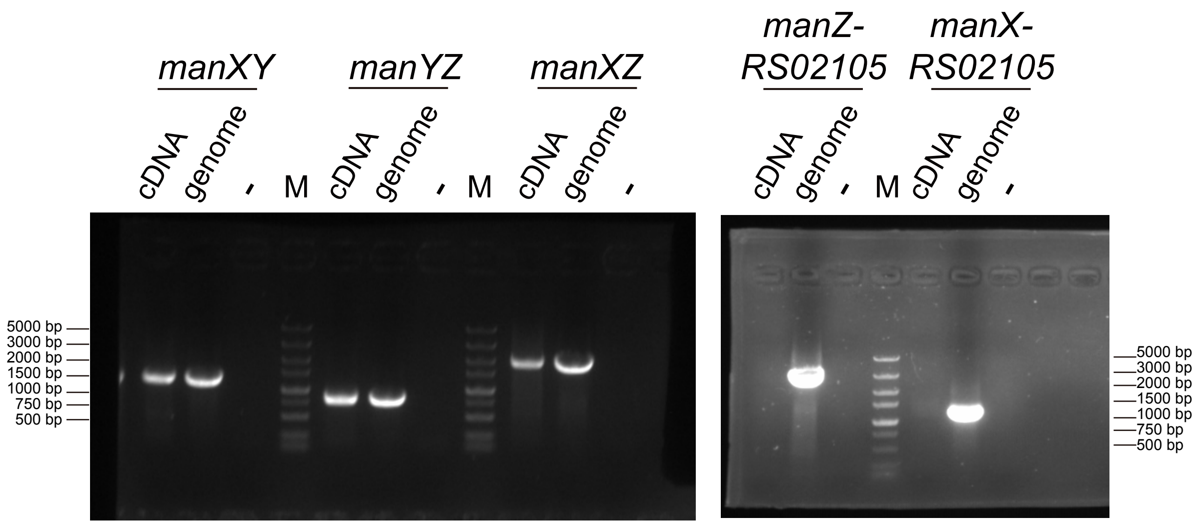

Extended Data Figure 7b

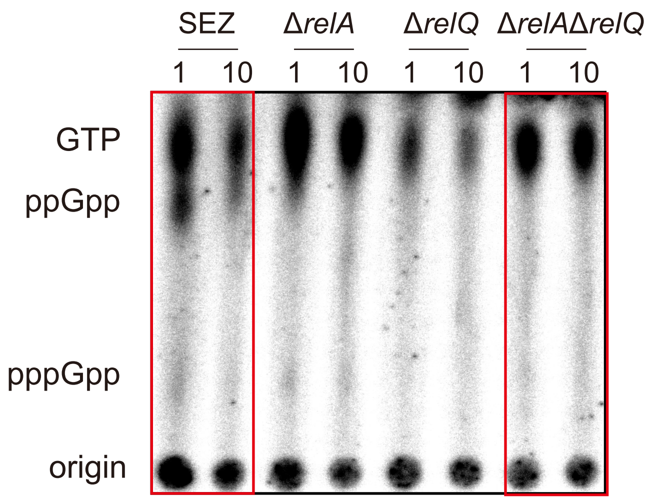

Supplement: Supplementary file 5 — Unprocessed blots or gels. [file 41564_2025_2194_MOESM5_ESM.pdf]
